# 2254: iobio: From academic project to commercial enterprise

**DOI:** 10.1017/cts.2017.141

**Published:** 2018-05-10

**Authors:** Alistair N. Ward, Chase Miller, Gabor Marth

**Affiliations:** The University of Utah School of Medicine, Salt Lake City, UT, USA

## Abstract

OBJECTIVES/SPECIFIC AIMS: The iobio project enables anyone (eg, diagnosticians, MDs, genetic counselors, citizen scientists) to perform useful analysis of genomic data, without a need to rely on bioinformaticians. iobio uses a novel real-time analysis framework, coupled with powerful visualizations delivered in a standard web browser. The project successfully supports free academic/nonprofit users, but occasions exist where it is more applicable for the project to be delivered commercially. Frameshift Genomics is developing commercial applications and functionality, which will exist alongside and in coordination with the academic project. These products will be marketed to large institutions including genome institutes, hospitals, diagnostic labs etc., but also to individual users who do not have access to large compute resources, or bioinformatic analysts, and everything in between. METHODS/STUDY POPULATION: The commercial iobio project under Frameshift Genomics aims to develop applications and features that cannot be successfully supported by an academic model. For example, when analyses are scaled up to processing of extremely large data sets, a commercial product with access to compute resources makes more sense than an academic tool. Bam.iobio is an application that samples data from sequencing alignment files, taking seconds to generate and visualize statistics representative of the entire file. This app is offered for free academically. When analysis involves thousands of such files, however, the commercial application, multibam.iobio, is more suitable. Other examples, including support for licensed third-party software and permitting extensive computation via cloud platforms, can also only be reasonably be supported via commercial software. Finally, development of commercial applications is driving adoption of more rigorous testing platforms, delivering more robust products. A particular strength of the iobio platform is allowing non-bioinformaticians to understand their data, for example providing quality control functionality providing confidence in data sets and the conclusions drawn from them. Such analyses are critical to all users of genomic data, and the iobio platform is ideally suited to provide an intuitive, integrated framework for performing them. RESULTS/ANTICIPATED RESULTS: The iobio project has been readily adopted by many in the community and shows significant promise for democratizing genomic analysis. Work is ongoing, supported by NIH small business grants, to develop commercial applications that will be marketed to analysts and medical professionals from large genome institutes and universities, to individual project users and citizen scientists. DISCUSSION/SIGNIFICANCE OF IMPACT: There are currently a number of iobio tools available academically, and they have been embraced by many in the genomics community. In fact, a number of popular platforms (eg, Galaxy, the International Cancer Genome Consortium (ICGC) data portal, mygene2 at the University of Washington) have incorporated iobio tools into their own platforms. To date, the gene.iobio variant interrogation tool has been used in a number of diagnostic projects, aiding identification of putative causative variants, and the pre-release version of the commercial multibam.iobio tool has been critical in unearthing data quality problems in project level data.

